# Trend in creatinine determining methods: Conventional methods to molecular‐based methods

**DOI:** 10.1002/ansa.202000074

**Published:** 2020-10-20

**Authors:** Ramin Narimani, Mahdad Esmaeili, Seyed Hossein Rasta, Hamid Tayebi Khosroshahi, Ahmad Mobed

**Affiliations:** ^1^ Medical Bioengineering Department, School of Advanced Medical Sciences Tabriz University of Medical Sciences Tabriz Iran; ^2^ Molecular Medicine Research Center Tabriz University of Medical Sciences Tabriz Iran; ^3^ Department of Medical Physics, School of Medicine Tabriz University of Medical Sciences Tabriz Iran; ^4^ Department of Biomedical Physics, School of Medical Sciences University of Aberdeen Aberdeen UK; ^5^ Center for Chronic Kidney Disease Tabriz University of Medical Sciences Tabriz Iran; ^6^ Department of Internal Medicine, Imam Reza Hospital Tabriz University of Medical Sciences Tabriz Iran; ^7^ Biotechnology Research Center Tabriz University of Medical Sciences Tabriz Iran; ^8^ Aging Research Institute Tabriz University of Medical Sciences Tabriz Iran

**Keywords:** biomarker, biosensor, electrochemical, enzyme, molecular imprinted polymer, nanoparticle

## Abstract

Renal failure (RF) disease is ranked as one of the most prevalent diseases with severe morbidity and mortality. Early diagnosis of RF leads to subsequent control of disease to reduce the poor prognosis. The level of sera creatinine is considered as a significant biomarker for kidney biofunction, which is routinely detected by the Jaffe reaction. The normal range for creatinine in the blood may be 0.84‐1.21 mg/dL. Low accuracy, insufficient sensitivity, explosive and toxicity of picric acid, and pseudo‐interaction with nonspecific elements such as ammonium ions in the Jaffe method lead to the development of various techniques for precise detection of creatinine such as spectroscopic, electrochemical, and chromatography approaches and sensors based on enzymes, molecular imprinted polymer and nanoparticles, etc. Based on previously established results, they are trying to construct sensors with high accuracy, optimum sensitivity, acceptable linear/calibration range, and limit of detection, which are small in size and applicable by the patient him/herself (point‐of‐care testing). By comparing the results of research, a molecularly imprinted electrochemiluminescence‐based sensor with linear/calibration range of 5‐1 mM

concentration of creatinine and the detection limit of 0.5 nM has the best detectable resolution with 2 million measurable points. In this paper, we will review the recently developed methods for measuring creatinine concentration and renal biofunction.

**Abbreviations**
CKD: chronic kidney disease
GFR: glomerular filtration rate
ISFET: ion‐selective field‐effect transistor
LOD: limit of detection
MIP: molecularly imprinted polymer
NPs: nanoparticles
POCT: point‐of‐care testing
SCr: serum creatinine

## 1 INTRODUCTION

Chronic kidney diseases (CKDs) are classified as the most prevalent diseases worldwide with severe morbidity and mortality, which causes a high economic burden on the healthcare system.^1^, ^2^, ^3^ Global studies indicate that the prevalence of CKDs is between 11% and 13%.^4^, ^5^ Kidneys are responsible for purifying systemic blood waste products, especially nitrogenous‐based waste products. In patients with renal failure, depending on the stages of failure, the waste products (creatinine and urea) can accumulate in the body. Creatinine (2‐amino‐1‐methyl‐2‐imidazoline‐4‐one) is the final metabolic product of creatine in muscle,^6^, ^7^, ^8^, ^9^ which is converted to creatinine approximately 2% daily at a constant rate.^10^, ^11^ The conversion is spontaneous and irreversible, which finally is excreted through the urine flow.^7^, ^12^ Creatinine is a nontoxic substance with no significant role in biometabolism with controlled concentration by renal excretion.^13^, ^14^ The amount of creatinine in the serum and urine is related to muscle mass and renal elimination, which is relatively stable in serum.^15^ Creatinine filtration is performed in the kidney without reabsorption.^6^, ^16^ Therefore, all of the creatinine produced by the contraction of the muscles is extruded from the body through the urinary tract.^17^, ^18^ The serum concentration of creatinine is an important biomarker for kidney failure detection, glomerular filtration rate, and muscular dystrophy.^13^, ^14^, ^15^, ^19^

Significant increases in the concentration of serum‐derived urea and creatinine (urea: as a reference for low‐molecular mass toxic solutes; creatinine: as a reference for toxic molecules of intermediate size), which are metabolic waste products of protein metabolism, are important markers for kidney dysfunction.^1^, ^20^, ^21^ Serum urea and creatinine concentration may raise up to 10 times of normal value in acute and also chronic renal failure conditions.^1^ Thus, the concentration of creatinine (molar mass: 113.12 g/mol) increases from 40‐150 μM (0.5–1.0 mg/dL for women and 0.7–1.2 mg/dL for men) to 1‐1.4 mM^22^, ^23^, ^24^, and for urea, the concentration increases from 1.7‐8.1 mM to 50‐70 mM ( 15‐50 mg/dL to more than 150 mg/dL)^25^, ^26^ (depending on gender and different age groups^27^). Therefore, measuring the creatinine and urea concentration with a faster, more accessible, cost‐benefit, and accurate method results in earlier diagnosis approaches and optimum management of patients with kidney disease.^28^

The most common method for measuring creatinine levels in serum and urine is based on Jaffe's reaction, in which creatinine changes in alkaline medium and responds with picric acid and turns into orange. This color change can be interpreted and evaluated.^6^ The Jaffe method, which was presented by Jaffe 130 years ago, is still used for its simplicity and low cost.^29^, ^30^ After Jaffe, given the limitations available for measuring creatinine, such as sample access, interference, reaction time, linear/calibration range, response time, sample magnitude, and large laboratory tools, various methods have been proposed, including enzyme reaction,^31^, ^32^ colorimetry assays,^33^, ^34^, ^35^ chemiluminescence,^36^ chromatography,^37^ molecularly imprinted polymer (MIP),^38^, ^39^ capillary electrophoresis,^40^ spectrophotometry methods,^17^, ^41^ potentiometric sensors,^42^ electrochemical sensors,^39^, ^43^ pH meters,^44^ and amperometric sensors.^45^, ^46^ These techniques are mostly performed on blood samples, urine specimens, and even saliva samples, which are referred to in various articles.^47^ It has also been observed that the results are improved by adding nanoparticles (NPs) to creatinine measuring methods.^48^, ^49^, ^50^, ^51^, ^52^

Along with creatinine, other markers have been utilized to determine kidney function. Some new biomarkers can identify renal diseases earlier.^53^, ^54^, ^55^, ^56^ The use of creatinine for the diagnosis of kidney function has limitations such as age, sex, race, and body weight, and therefore, there is a need for better markers that some of them are now used as practice. Glomerular filtration rate (GFR) is a measurement unit used to determine the amount of blood passing through the kidneys per minute and its amount can be indicative of kidney function.^36^ In measuring GFR, factors such as age, body size, weight, race, and gender are involved in the final stage. Evidence suggests that GFR is one of the best indicators of renal function in CKD. For measuring GFR, urine isotope collections such as inulin, iothalamate, ethylene diamine tetraacetic acid, and iohexol are considered as gold standards, however, they are invasive, impractical, time consuming, and expensive in the clinical setting. Also, this method cannot account for individual differences in muscle mass for a specific age, gender, and race.^57^, ^58^

Urea is another indicator of kidney function that can be detected in blood, urine, and saliva. For this purpose, various sensors have been designed and built. One of them is the colorimetric detection of urea in urine by Deng and colleagues.^59^ Lee et al and Silva et al also developed the high sensitive ion‐sensitive field‐effect‐transistor (ISFET) biosensors for urea with acceptable precision.^60^, ^61^ Recently, a portable, low‐cost, and easy to use urea biosensor for patient care was also designed by Wang et al with detection range of 24‐300 mg/dL.^62^ Whenever the amount of protein metabolism increases or the activity of the kidneys gets disturbed or the blood becomes thick, the number of urea increases. Therefore, this test is mainly used to check the body's water and to some extent the activity of the kidneys.

Also, cystatin C is a promising biomarker for early detection of renal injury. Its production rate is relatively constant from 4 months to 70 years and is proportional to GFR. Unlike serum creatinine (SCr), its production is not affected by muscle mass, sex, and race. Because of its small size and positive net charge, it is freely filtered in the glomerulus. Cystatin C is catabolized and reabsorbed completely, with a lack of tubular secretion in the proximal tubule.^57^, ^63^ Changes in serum cystatin C are used as an indirect measure of GFR. Nowadays, due to the expensive and unavailable materials for measuring cystatin C, this test is not routinely performed in laboratories. Different diagnostic methods and sensors for cysteine C have been proposed, which can be referred to as a novel photoelectrochemical immunosensor by the integration of nanobody and TiO_2_ nanotubes developed by Mi et al,^64^ and an ultrasensitive protein‐protein interaction–based SPMWE sensor designed by Desai et al.^65^

The purpose of this study is to develop a simpler measuring method for creatinine assay with high accuracy, reasonable cost, faster, and reliable result that can be used in emergency departments, medical centers, and small clinics, especially in urban areas.^66^, ^67^ In this article, we review the methods of measuring creatinine concentration, and expressed the advantages and disadvantages of each, and compare their detectable resolution.

## 2 METHODS IN THE DETERMINATION OF CREATININE

### 2.1 Chromatography

Chromatography is one of the most common and old methods for separating molecules of a mixture. The separated or extracted analyte can be examined by a spectrophotometer and other detectors. One of the most important chromatographic analysis devices, which can be used to separate, measure, and identify a variety of materials, is high‐performance liquid chromatography (HPLC). The HPLC method uses a mobile high‐pressure liquid phase to wash and displace the sample along the column and separate the compounds of a mixture. In one of the HPLC procedures, the creatinine is separated from the other species of blood serum by the cation‐exchange chromatography and the absorbance is measured in 234 nm.^68^ Zhao described the simultaneous measurement of kidney indices (creatinine, uric acid, kynurenine, and tryptophan). In this method (HPLC with ultraviolet [UV] detection method), an Agilent HC‐C18 analytical column has been used for separation. Limit of detection (LOD) and linear/calibration range of creatinine in this method are 0.1 μM and 20‐280 μM. The total run time in this method was 25 minutes.^37^ Also, in bovine plasma, a simple and rapid method for the determination of creatinine is described. Plasma was chromatographed for 15 minutes. Lowest detectable amounts (LOD) of creatinine were 0.28 nM in this method.^69^

Significant advances in instrumentation and column technology were made to significantly increase resolution (resolution), velocity, and sensitivity in liquid chromatography (LC), which resulted in the ultra‐high performance liquid chromatography (UHPLC). In UHPLC, to improve the efficiency of chromatographic separation, the particle size of the stationary phase has been reduced.^70^ Fraselle et al. have developed and validated an UHPLC tandem mass spectrometry (UHPLC–MS/MS) method for measuring creatinine in human urine. In this method, the linear range is between 0 and 500 mg/dL, with LOD of 0.5 mg/dL.^71^ UHPLC include very low detection limit (LOD), high sensitivity and selectivity, and the possibility of examining the sample in the presence of chemical disturbances.

A LC‐MS method is also developed. This method has a faster analysis rate than other chromatographic methods. The accuracy and speed of analysis in this method is more than other methods of chromatography (less than 10 min) ^90^. Two‐dimensional LC (2D‐LC) is defined as the technique in which two liquid phase separation systems (independently) are applied to a sample. 2D‐LC has received a great deal of more attention over the past few years because of its high resolving power, especially for dealing with complex samples.^72^ For this purpose, Eggink et al. have developed and optimized a comprehensive 2D‐LC‐based system with UV detection and MS for the separation of complex specimens by multistep gradient elution with the ability to detect creatinine.^73^

### 2.2 Enzymes‐based assay

**FIGURE 1:**
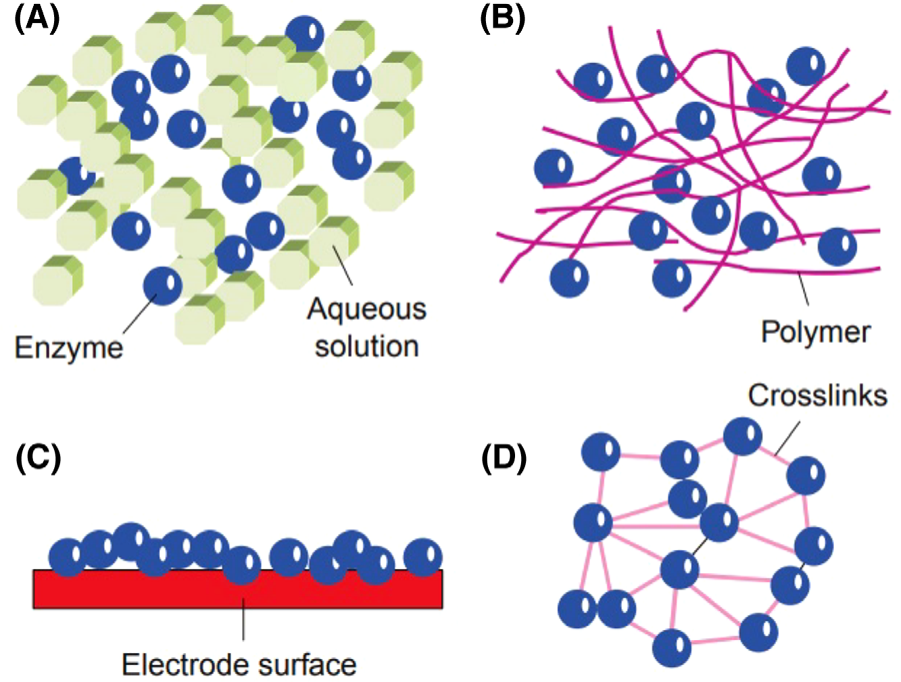
Simple methods of enzyme immobilization engaged in creatinine biosensors. (A) Gel entrapment marks in the aqueous environment and enzyme being trapped. (B) Enzymes can be trapped within growing polymers. These can be both nonelectroactive and electroactive combinations of polymers. (C) Inactive, noncovalent connections with electrode surfaces have been used, but are unpredictable. (D) Cross‐linking of enzymes results in major stability but this is often at the expense of enzyme activity and sensitivity.^75^

Enzymes are protein structures that can have a special catalytic role. They are not consumed in chemical reactions and do not change the balance of a reaction. Enzymes can also have the role of locks and keys for a specific substrate and only affect specific molecules.^74^ Here, creatinine acts as a substrate, and the task of enzymes is to convert creatinine into measurable material (without interfering with other substances); also enzymes are reactivated with the substrate in a place called the active site (under certain environmental conditions in terms of temperature and pH, etc.). Simple methods of enzyme immobilization engaged in creatinine biosensors are shown in Figure 1.^75^

Depending on environmental conditions including temperature and pH, high cost of their purification and production,^76^, ^77^ enzyme concentration, ionic strength,^78^ the concentration of the substrate, and lifetime (enzyme activity) are the limitations of the enzymes used as bioassay, some of which is repaired by modifying the structure and immobilization method.^77^, ^79^

Some of the enzymes used in the creatinine sensor, for decomposing creatinine into measurable materials, are as follows:


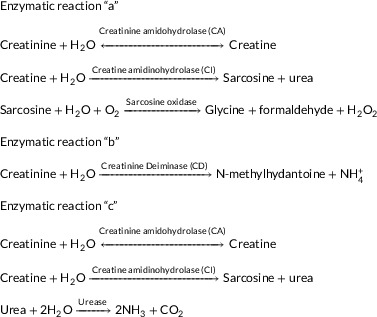




In the reaction of the three enzymes, in reaction “a,” the $H_{2}O_{2}$ obtained from the reaction is used by amperometric biosensors to detect creatinine.^75^, ^80^, ^81^ Oxygen detector electrodes are also used for this reaction.^82^ In reaction “b,” the ${\mathrm{NH}}_{4}^{+}$ obtained from the reaction is used as a transducer to detect creatinine concentrations (potentiometric biosensors and pH sensors,^83^ etc.).^75^ The simplicity of this reaction, which is used only by an enzyme, is one of the advantages of this method. One of the problems is the endogenous NH4+ interference with blood, and in particular with the urine sample.^75^ Reaction “c” is also used to construct potentiometric creatinine biosensors. The complexity of the three‐enzyme systems and the reduced sensitivity of these systems due to the presence of enzymes, and also dual systems that separate creatine from creatinine, have affected the development and construction of these sensors.^66^, ^75^, ^78^ The term of the enzyme by attachment to the inert and forming a distinct solid matrix is called enzyme immobilization. The method of enzyme immobilization in both three‐enzyme and single‐enzyme creatinine biosensors is the most imperative factor in the determination of their analytical range, and their effective and storing stability. Since stability is one of the critical challenges with these systems, several methods have been examined to find ways of constructing sensors with commercial potential. These have involved direct nonimmobilized deposition,^84^ gel entrapment,^85^ cross‐linking,^86^ polymer entrapment,^87^ and mixtures of these methods. Another system was developed some form of cross‐linking, which permits developed stability typically at the expense of reduced sensitivity and reduced analytical range.^75^

### 2.3 Nano‐based methods in determination of creatinine

Along with the development of nanotechnology and the creation of nanoscale materials, various biosensors are also designed and constructed. Different syntheses of a substance can cause the shape and size (adjustable) in an NP (changing the physicochemical properties) that can be used for various applications.^88^, ^89^ NPs are classified according to their shape, size, and properties into various groups, including ceramic, metal, polymeric, and fullerenes NPs.^90^, ^91^ The use of NPs has also been developed to determine the concentration of creatinine and early diagnosing of CKD.^92^ The high ability of surface reaction, large S‐V (surface‐to‐volume) ratio, high absorption ability, and improved catalysis are the characteristics of nanomaterials, which makes it possible to use them in biosensors.^47^, ^93^ Nanomaterials are used in electrochemical electrodes due to the ability to increase the electron transfer rate between the active site of the enzyme and the electrode.^94^ Also, the change in absorption and color obtained by adding creatinine to a solution containing NPs is used in detecting creatinine concentration.^35^, ^49^, ^95^ Du et al. have created a synergistic coordinating system of creatinine/adenosine on an AuNP surface with Ag+ that has a quick, selective, and quantitative detection of creatinine. Because of their unique synergistic coordination capability to coordinate adenosine and creatinine with Ag+ on a particle surface, they used a colorimetric sensor based on AuNPs to determine creatinine. There are linear relationships of good absorption changes to creatinine concentration, so both qualitative detection of colorimetry with the naked eye and quantification by UV‐Vis spectrometer (A630 nm/520 nm) can be achieved. This system has a linear/calibration range of 0.2‐1.4 μM and LOD of 12.7 nM.^95^ Some of the NPs that are used in the electrochemical and colorimetric methods to detect creatinine, because of the properties of NPs described above, are given in the Table 1.

**TABLE 1:** Some of the used nanoparticles in the electrochemical and colorimetric methods to detect creatinine

Methods	Nanoparticles	Linear/calibration range	LOD	Sensitivity $\left(\mu A\;\mu M^{-1}{\mathrm{cm}}^{-2}\right)$	Refs.
Amperometric	ZnO‐NPs/CHIT/c‐MWCNT/PANI composite film	10‐650 μM	0.5 μM	0.030	^94^
Amperometric	c‐MWCNT/PANI	10‐750 μM	0.1 μM	40	^96^
Amperometric	Gold nanoparticles (AuNPs), multiwalled carbon nanotubes (MWCNTs) and Teflon	3‐1000 μM	0.1 μM	–	^50^
Amperometric	Novel ammonium ion‐specific copper‐polyaniline nanocomposite	1‐125 μM	0.5 μM	85 ± 3.4	^1^
Amperometric	Iron oxide nanoparticles/chitosan‐graft‐polyaniline (Fe3O4‐NPs/CHIT‐g‐PANI) composite film	1‐800 μM	1 μM	3.9	^97^
Spectrophotometry	Gold nanoparticles (AuNPs)	15‐40 $\mathrm{mg}.L^{-1}$ (132.6‐354 μM)	13.7 $\;mg.L^{-1}$ (121 μM)	A600/520	^98^
Spectrophotometry	Silver nanoparticles capped with 2,2‐thiodiacetic acid	0.01‐1 μM	3 nM	A560/A390	^49^
Colorimetric	AuNPs	0.2‐1.4 μM	12.7 nM	A630/520	^95^

### 2.4 MIP method

**FIGURE 2:**
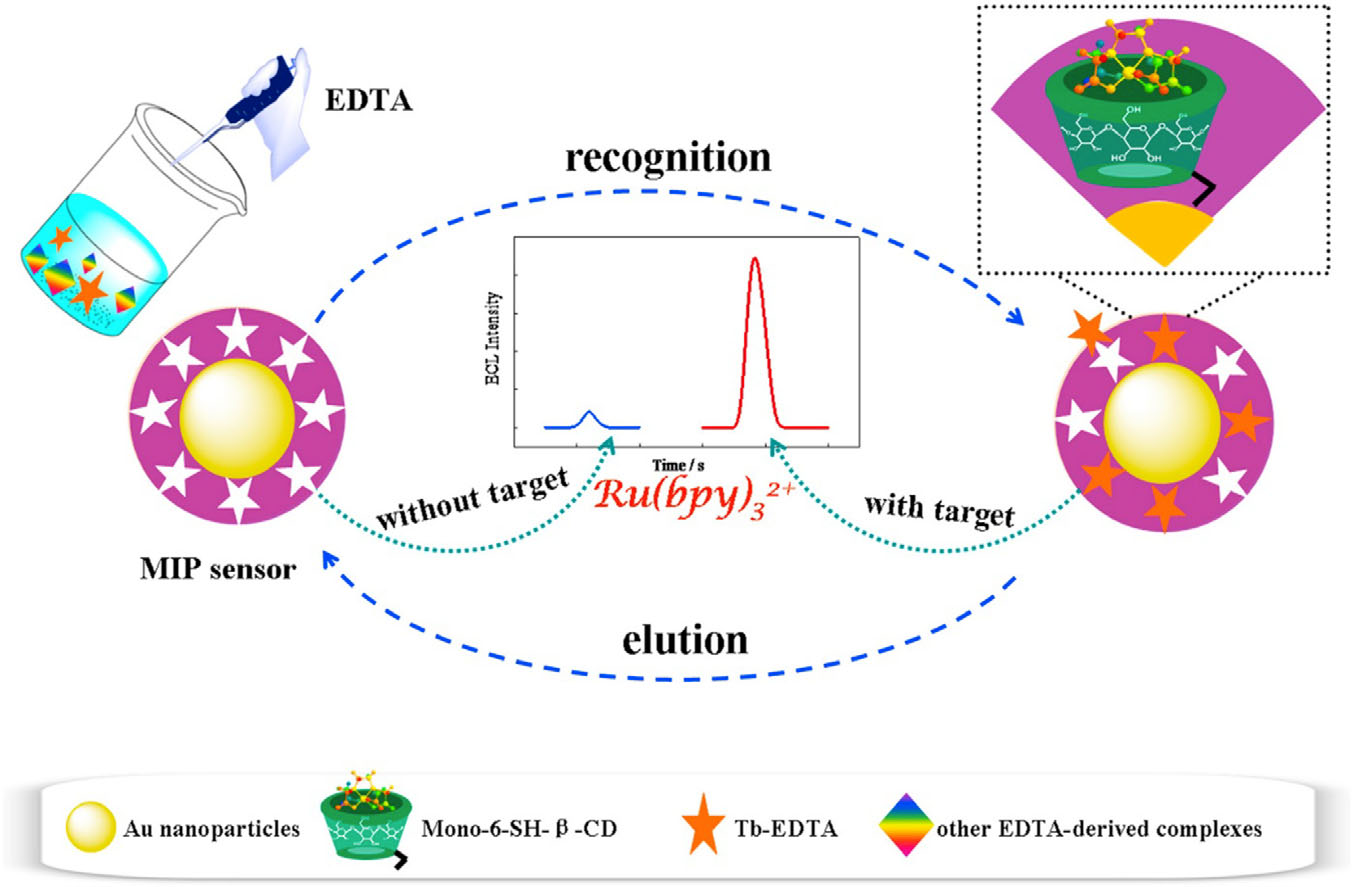
Schematic of the function of the MIP method.^109^

Molecular imprinting is a technique to create template‐shaped cavities in polymer matrices with predetermined selectivity and high affinity. This technique is based on the system used by enzymes for substrate recognition, which is called the “lock and key” model,^99^ which is shown in Figure 2. This technique acts as an artificial receptor bioanalyst.^14^, ^100^ Choosing the functional monomer for complementary interactions with the substrates and template molecule is very important. The complete removal of the template from the polymer is one of the main problems of this method.^99^, ^101^, ^102^ After the removal of the template, the target molecules get involved with the cavities and measured by impedimetric,^103^, ^104^ capacitance,^105^ optical,^106^ amperometric,^107^ and chromatographic methods.^108^

**FIGURE 3:**
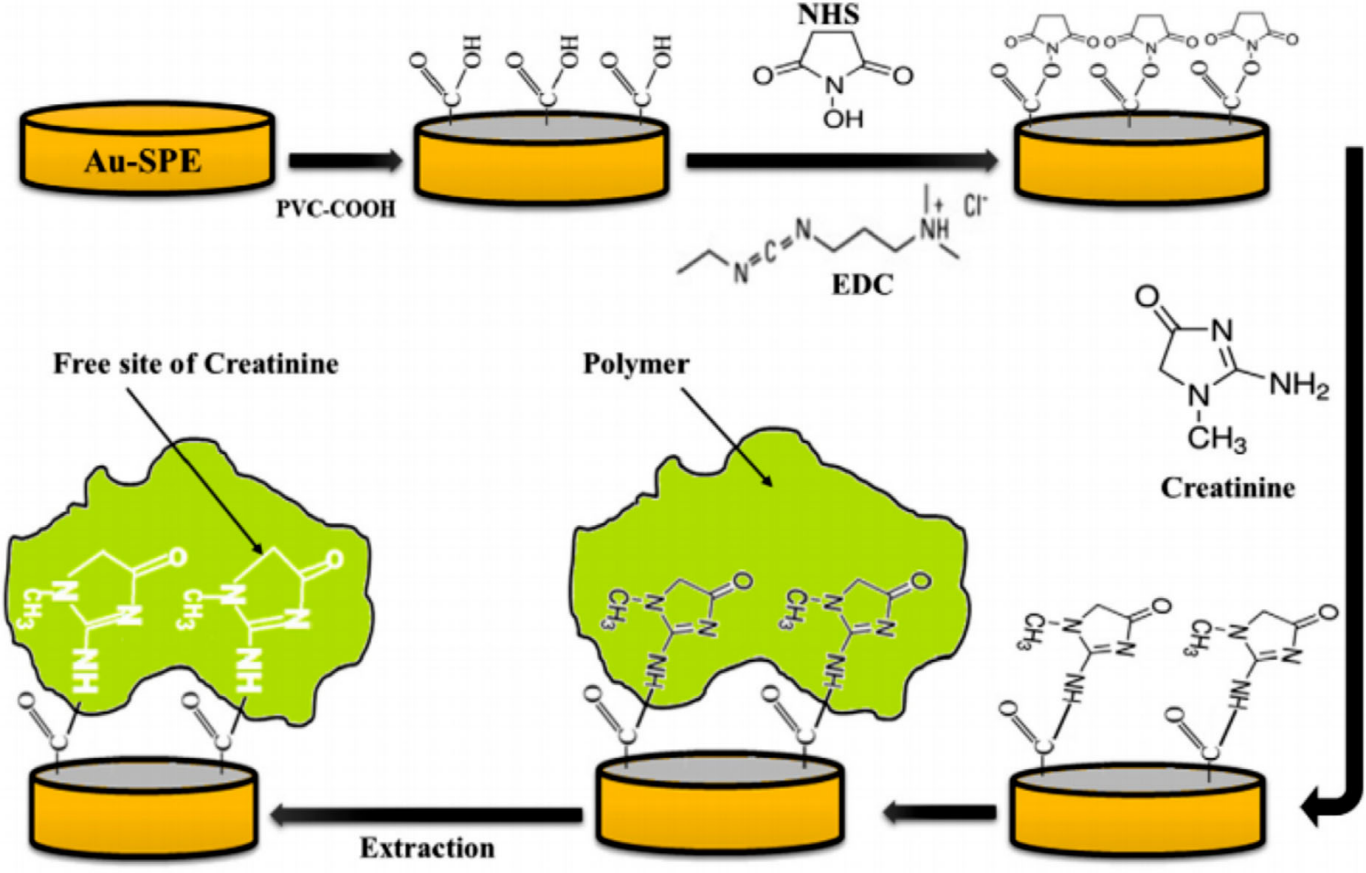
Schematic representation of Au‐SPE/MIP procedures.^38^

**FIGURE 4:**
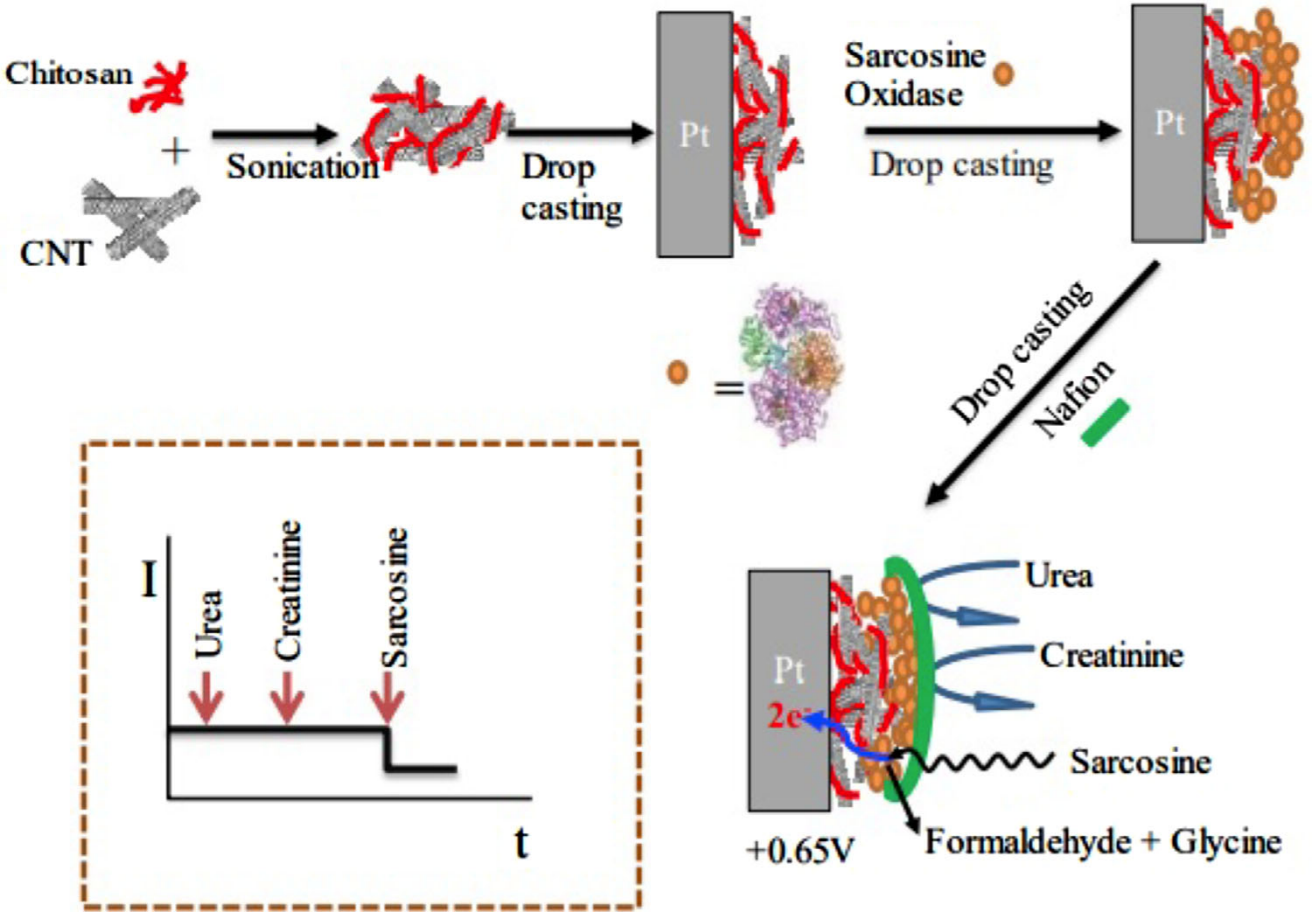
Schematic representation of the LbL sarcosine electrochemical biosensor and idealized current time response toward analyte and interferents.^110^

In the MIP method, the cavities in the polymer structure have a specific property in terms of size, shape, chemical, and physical properties. Therefore, this method has a higher sensitivity, stability, and selective property. Also, due to its low cost and relative simplicity, this method has been used more recently.^108^ A novel biosensor based on MIP was developed for the detection of creatinine in humane urine using screen‐printed gold electrodes (Au‐SPE). In this method, a layer of polyvinyl carboxylic chloride (PVC‐COOH) was deposited on the Au‐SPE surface. Creatinine molecules were attached to the Au‐SPE/PVC‐COOH surface. Subsequently, polymerization of acrylamide and N,N‐methylene bisacrylamide filled the void around them. Subsequent patterns remove binding sites within the polymer that can selectively detect creatinine at different concentrations. Their retention properties and molecular identification were qualitatively investigated using three instrumentation techniques: voltammetry, electrochemical impedance spectroscopy, and spectrophotometry. The simplicity of operation, highly selective recognition ability, low cost, and small size are remarkable in this platform (Figure 3).^38^ The functional layer–by–layer (LbL) electrochemical system was established successfully for the effective detection of creatinine (Figure 4). This work presents a functional LbL electrochemical biosensing scheme for the effective detection of sarcosine as well as a functional component of a creatinine sensor; both of these systems can be used for multiple clinical diagnoses. The LbL approach shown in this study allows overcoming the unique shortcomings of materials, such as the lack of chitosan conductivity and the lack of dissolution of carbon nanotubes. The performance of our sensing system is comparable to other electrochemical designs, indicating that the system has a linear detection limit across protection relationships, durability for real clinical applications, selectivity against interfering molecule, and successful performance in body fluids. Developed systems was applied to several clinical analyses approach and can be improved to microelectrodes for the real‐time procedure at the patient's bedhead.^110^ Also, Han et al. have developed a modified electrode based on phosphotungstic acid using the LbL method. In this method, an electrode was used to determine creatinine directly with the assistance of copper II. The quantity of creatinine was determined by measuring the redox peak current of Cu(II)‐creatinine complex/Cu(I)‐creatinine complex. The linear range and the detection limit are 0.125‐62.5 μM and 0.06 μM, respectively.^111^ Some MIP methods for detecting creatinine are listed in Table 2.

**TABLE 2:** Some monomers and cross‐linkers that are used in MIP

Methods	Monomers	Cross‐linkers	Linear/calibration range	Limit of detection	Refs.
Electrochemical impedance method	Poly ethylene‐co‐vinyl alcohol (EVAL)	Ethanol solution of 1‐octadecanethiol	0.05‐2 μg/mL (0.44‐17.68 μM)	40 ng/mL (0.35 μM)	^112^
Electrochemical voltammetric biosensor	Polyoxometalate functionalized reduced graphene oxide (rGO)	Ethanol solution	0.05‐1.5 nM	0.0151 nM	^113^
Spectrophotometer (Langmuir and Freundlich adsorption isotherm models)	N‐Methacryloyl‐(L)‐ histidinemethylester (MAH)	Ethanol solution	0.1‐ 2.0 mg/mL (0.884‐17.68 mM)	–	^114^
Economic electrochemiluminescence	Bovine serum albumin	tetraethyl orthosilicate (TEOS)	5 nM‐1 mM	0.5 nM	^115^
Electrochemical	Aniline		20‐1000 nM	0.35 nM	^116^
Electrochemical impedance analysis	Methylacrylate	Ethylene glycol dimethacrylate	20‐670 ng/mL^−1^ (0.18‐5.92 μM)	20 ng mL^−1^ (0.18 μM)	^103^
Capacitive	2‐Acrylamido‐2‐methy l‐1‐propanesulfonic acid (AMPS)	N, N‐Methylenediacrylamide (MBA)	100‐600 μM	10 μM	^105^
Chromatography	Poly ethylene‐co‐vinyl‐alcohol (EVAL) and 4‐vinylpyridine (4‐Vpy)	Divinylbenzene	–	–	^117^
The immobilized enzyme membrane	Poly(y‐methyl‐L‐glutamate)		1‐10 $\mathrm{mg}.dL^{-1}$ (88.4‐884 μM)	0.5 $\mathrm{mg}.dL^{-1}$ (44.2 μM)	^118^
Cathodic stripping voltammetric	Melamine (mel) and chloranil (chl)	Low level cross‐linking network	0.0025‐84.0 μg. $mL^{-1}$ (0.022‐742 μM)	1.49 ng. $mL^{-1}$ (0.013 μM)	^14^

### 2.5 Electrochemical methods

The relationship between chemical changes and electrical energy is important (theoretically and practically). Electrochemistry is a science that is related to electricity on the one hand, and to chemistry on the other hand, and what connects these two sciences is an electron (electricity flow). In electrochemical methods, the analyte is usually converted to a measurable substance. This is usually done by enzymes, MIP, or using NPs. Some of the electrochemical methods that are used to make biosensors are given below.

#### 2.5.1 Amperometric biosensors

Measuring the current in a chemical reaction (with electrodes) that results from oxidation or reduction is the principle of working with amperometric methods. In the amperometric methods, three electrodes are used: working electrode, reference electrode (mostly Ag/AgCl), and counter (auxiliary) electrode (inert metal).^119^ In amperometric techniques, single‐enzyme^120^, ^121^ and three‐enzyme^81^ methods are used commonly.^80^, ^119^, ^122^ The need for high potential for reducing oxygen (in DO‐meter sensors) and use of three‐enzyme systems due to the complexity and loss of sensitivity and also the use of dual sensors to separate creatine/creatinine are the disadvantages of this method. Antibodies are also used as receptors in amperometric sensors. The use of electrochemical methods along with immunology is called immunosensors. A specific antigen or antibody is immobilized on the surface of electrode, and by reacting with an analyte, the concentration of the target substance is detected.^123^ Some results from a variety of amperometric sensors are given in Table 3.

**TABLE 3:** Some results from a variety of amperometric sensors

Type of diagnosis	Structure of the electrode (immobilization method)	Enzymes	Linear/calibration range	LOD	Sensitivity $\left(\mu A\;\mu M^{-1}{\mathrm{cm}}^{-2}\right)$	Refs.
H_2_O_2_	Poly(ethylene glycol) diglycidyl ether (PEGDGE)/glassy carbon (GC)( cross‐linking)	Three‐ enzymes/ POD/ PVI[Fe(CN)_5_]	12‐500 μM	12 μM	11 000	^126^
H_2_O_2_	Platinum working electrode/ creatinine‐modified membrane (cellulose membranes/ carbonyldiimidazole)	Monoclonal antibodies (hybridoma technology)	0.01‐10 μg/mL ( 0.088‐88.4 μM)	4.5 ng/mL (40 nM).	–	^49^
NH_4_+	Nafion®‐nanostructured polyaniline (nsPANi) composite	Creatinine deiminase (CD)	1‐125 μM	0.5 μM	1300	^128^
–	Copper‐deposited electrode	Enzyme‐free	0.025 ‐1.5 mg/dL (2.2 ‐132.6 μM)	6.8 μg/dl (0.6 μM)	–	^167^
H_2_O_2_	Enzyme/c‐MWCNT/PANI/Pt ( covalent)	Three‐ enzymes	10‐ 750 μM	0.1 μM	40	^60^
H_2_O_2_	Poly(acrylic acid)/ argon‐plasma‐treated porous polypropylene (graft polymerization)	Three‐ enzymes	3.2‐320 μM	–	–	^166^
–	ZnO‐NPs/CHIT/c‐MWCNT/PANI/Pt	Three‐ enzymes	10‐650 μM	0.5 μM	0.03	^113^
–	Anti‐creatinine antibodies/ redox‐labeled creatinine/ glassy carbon electrode	Size exclusion redox‐labeled immunoassay (SERI)	0.09‐900 μM	400 nM	–	^165^
–	Poly methylene blue (PMB)‐Cu‐Carbon nanofiber (CNF) nanocomposite	Enzyme‐free	0.5‐900 ng/mL (4.42‐7956 nm)	0.2 ng/mL (1.77 nM)	0.133 μA ng mL^−1^	^37^

#### 2.5.2 Potentiometric biosensors

**FIGURE 5:**
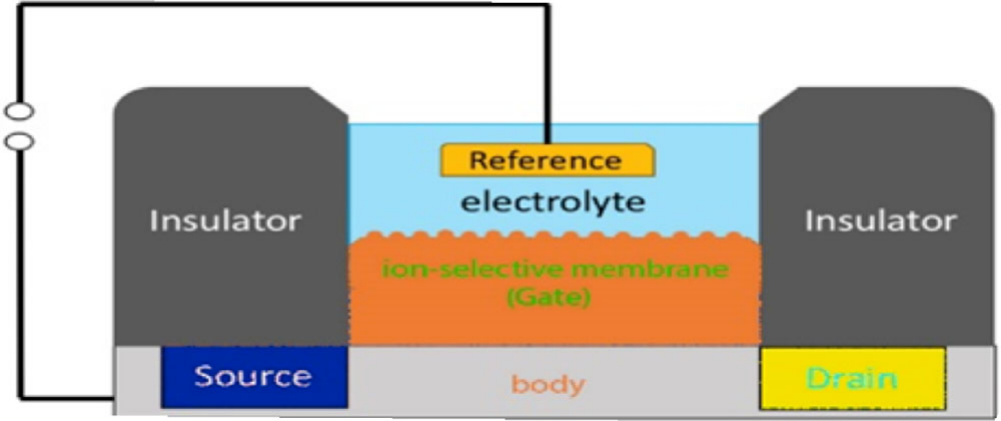
The schematic view of an ISFET: concentration gradient of charged analyte ions creates a chemical potential between the source and gate, which is in turn measured by the FET.^128^

The measurement of the potential difference between the working electrode and the reference electrode in an electrochemical cell (when the current flow is zero or very small) is used in potentiometric biosensors. Some of the potentiometric creatinine sensors are based on enzymes (hydrolysis of creatinine), that sensors work on the detection of pH (measure the liberated hydrogen ions by enzymatic hydrolysis) and ammonium ion detectors (liberated ammonium ions formed by enzymatic hydrolysis).^85^ In 1976, the first potentiometric creatinine biosensor was introduced by Meyerhoff and Rechnitz using an ammonia sensitive electrode.^124^ Since then, enzymatic and MIP‐based electrodes for creatinine have been developed. Stability is one of the problems of enzymatic systems. Different methods of immobilization have been investigated to solve this problem and build stable and economically sensors including covalent, cross‐linking, entrapment, and adsorption. In most potentiometric methods, Ag/AgCl is used as a reference electrode.^42^, ^125^ One of the new potentiometric methods is the use of a particular type of FET‐based biosensors in which the metal‐gate electrode has been replaced with an ion‐selective membrane, electrolyte, and a reference electrode (ISFET). Among them, certain types that are immobilized with enzymes are called enzyme‐sensitive FET. The MIP is also used to build ISFET‐based biosensors. It is used to make creatinine‐sensitive ions that replace the gate terminal.^126^, ^127^ The algorithm of the ISFET method is illustrated in Figure 5.

The results of measuring creatinine by potentiometric techniques are presented in Table 4.

**TABLE 4:** The results of the potentiometric techniques for the detection of creatinine

Workingelectrode	Structure of the electrode (immobilization method)	Enzymes	Linear/calibration range	LOD	Sensitivity ($\mathrm{mV}\;\mathrm{de}c^{-1}$)	Refs.
pH‐sensitive field‐effect transistors (pH‐FET)	Entrapment	CD	0.02‐2 mM	20 μM	40 mV/pH	^129^
pH‐FET	Adsorption	CD	0‐2 mM	5 μM	40 mV/pH	^130^
pH‐FET	Covalently cross‐linked	CD	0‐2 mM	10 μM	40 mV/pH	^131^
pH‐sensitive/ ammonium ion‐selective	SPCE/nonactin and bis‐(2‐etylhexyl) sebacate (DOS)(entrapment)	CIH	40‐140μM(ph) 15‐140 μM (ammonium)	26 μM (ph) 3 μM (ammonium)	29.45 (ph) 46.66 (ammonium)	^132^
Cyclic voltammetry	PEI/PTA multilayer film coated ITO	Enzymeless	0.125‐62.5 μM	0.06 μM	200 mV s^−1^	^111^
–	Sol‐gel film on graphite electrode	MIP	1.23‐ 100$\;\mathrm{mg}.L^{-1}$ (10.9‐884 μM)	0.37 $\mathrm{mg}.L^{-1}$ (3.3 μM)	–	^133^
–	Calix pyrrole molecule	Ionophore‐based ion‐selective electrode	1 μM‐10 mM	$10^{-6.2}$M	54.1 ± 0.6	^42^
Ammonium ion‐selective	Carboxylated poly (vinyl chloride)/ 1‐ethyl‐3‐(3‐dimethylaminopropyl) carbodiimide hydrochloride (EDAC) (covalent)	CD	0.02‐20.0 mM	15 μM		^134^
Ammonium ion selective	Tetrahydrofuran (THF)/chitosan (adsorption)	CIH	0.1‐10 mM	0.1 mM	50	^135^
Enzymeless	β‐cyclodextrin (βCD)/ poly‐3,4‐ethylenedioxythiophene (PEDOT)/ glassy carbon electrode (GCE) ( noncovalent)	–	0.1‐100 mM	0.05 mM	*–*	^136^
–	MIP‐TMSPMA‐GO‐co‐HEMA/MMA	MIP	0.5‐3.0 mg/dL (44.2‐265.2 μM)	0.1878 mg/dL (16.6 μM)	*–*	^137^

#### 2.5.3 Conductometric biosensor

**FIGURE 6:**
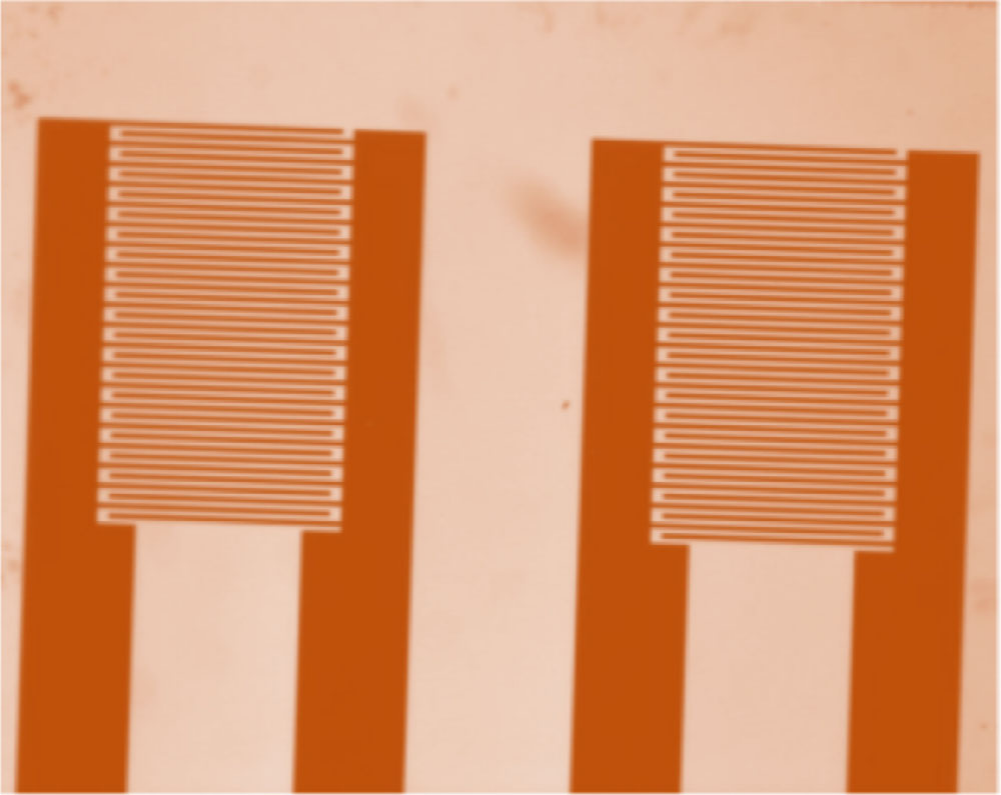
Two pairs of planar interdigitated electrodes (optical microscopy view of the conductometric sensor)

The electrical conductivity is directly related to the conductive salts in the liquid. If the amount of conductive salts increases, the electrical conductivity will increase. Conductometer is a device for measuring the electrical conductivity of fluids. The conductivity does not have the capability of measuring a specific ion in the sample and is used to estimate the overall ion content. Conductometric titration is used to determine the concentration of ions in the samples (by measurement of its effect on the electrical conductivity of the mixture). The lack of reference electrodes, high compatibility, lack of light sensitivity, and the ability to minimize the design of differential electrodes are the advantages of this type of sensor.^138^, ^139^ The use of the creatinine deaminize that has been entrapped on a PVA/PEI/AuNP composite film has been used to make a conductometric creatinine sensor. This method provides a large linear range and good LOD for determining the concentration of creatinine.^139^ The optical microscopy view of the conductometric sensor is shown in Figure 6. A conductometric creatinine biosensor based on solid‐state contact ammonium sensitive PVC‐NH 2 membrane is also provided with an acceptable linear range and LOD.^138^

#### 2.5.4 Capacitive/impedometric sensors

Capacitive/impedometric sensors are other types of electrochemical sensors, in which mostly MIP methods are used as dielectric.^105^, ^140^ The amount of analyte absorption of the receptors determines the measurement accuracy of the capacitor sensors. Therefore, capacitance sensors are based on changes in the effective thickness of the insulating layers, which results in a change in the analyte absorption that is located at the surface of the receptor of the capacitive sensor.^141^ Over the past decade, the use of capacitive detection has been used in the field of adsorption rate in classical electrochemistry.^142^ Capacitive detection in the field of immunosensors, biological and chemical sensors, and enzymatic biosensors is widely used. Capacitive sensors are also used to detect creatinine concentrations. The first creatinine sensors based on capacitive detection and artificial sensors had a good LOD. Also, creatinine, NaCl, and glucose did not interfere with these sensors. Molecularly imprinted photopolymerization of acrylamidomethylpropanesulfonic acid and methylenediacrylamide cover the receptor layer of this sensor. On the surface of the golden electrodes, covered with an alkynyl alcohol lacquer, a polymeric layer was grafted. The change in electrode capacitance indicates the amount of electrode capacitance and thus the concentration of creatinine is detected. As stated, the response of this chemosensor is reversible and highly selective.^105^ Label‐free sensing of creatinine has also been developed using a complementary metal oxide semiconductor (CMOS) near‐field dielectric immunosensor operating at 6 GHz (C‐band). In that study, a multi‐fingered planar interdigitated capacitor is used as the archetype capacitive sensor. In this way, creatinine molecules are immobilized on the surface of silicon nitride layers (Si_3_N_4_), which helps to evade any additional postprocessing for label‐free creatinine detection sensors. The linear/calibration range of creatinine is suitable in this method.^143^

Capacitive chemosensors necessarily require an ultrathin layer of MIP grafting over the solid electrodes. This factor increases the thickness of the film, which causes a deviation of about 10% in experimental results. Also, the use of real probes may cause deviation in capacitance data because the capacitive sensors involved electrode fouling risk. Signal changes in capacitor sensors are about a few percent, but the resulting signal of them has good stability, and therefore the signal‐to‐noise ratio is higher.^141^ There is no chemical reaction in capacitor sensors, and so the pH value does not affect the results.^140^ Response time is one of the main characteristics of electrochemical sensors. The response time for potentiometric sensors is about 4‐10 seconds and for amperometric sensors, it is about 14 seconds. This time for capacitive biosensors is about 2 minutes. Recently, a capacitive sensor has been developed in which a dentate shape is used to form the dielectric to improve the diffusion. The response time for this sensor is reported to be 0.036 second.^140^

### 2.6 Spectroscopic and colorimetric (optical) methods

According to research in the last 5 years, most of the analytical and biomatrices techniques described for creatinine are related to colorimetric methods.^144^ The reaction of creatinine with picric acid in an alkaline environment was performed by Max Jaffe in 1886. This reaction creates an orange color in the solution, which is measured by a spectrophotometer.^29^ A spectrophotometer measures the amount of light absorbed by a sample. One of the best results in the spectrophotometric method is the use of silver NPs coated with picric acid. The initial color of this solution is yellow, which changes to red with the addition of creatinine.^145^

Another type of spectroscopic technique is Raman spectroscopy.^146^ In Raman spectroscopy experiments, strong monochromatic photons (such as a laser beam) are concentrated on the sample. The Raman scattering is an inelastic process (each molecule has its characteristic Raman spectrum) that helps to determine the composition of the sample.^147^ It shows vibrational energy levels of the molecule's chemical bonds.^148^ Simple sample preparation, no need for markup methods, higher sensitivity, providing a wealth of information on the molecular fine structure, and the ability to achieve nondestructive testing of samples are the benefits of this method.^149^ Stosch et al. have developed a novel surface‐enhanced Raman scattering approach to quantify creatinine in human serum. Surface‐enhanced Raman scattering obtains the character of a ratio method that works similarly to the well‐established isotope dilution techniques, using isotopically labeled creatinine as an internal standard. The surface‐enhanced Raman scattering, compared to the Raman scattering, provides some additional spectral information that may be extracted and used to support the desired quantitative evaluation. This method was successfully used to quantify creatinine at clinically relevant and low levels, along with multivariate data analysis. The LOD for creatinine in this method was found less than 0.1 μg/mL, which can be reliably detected.^150^ Also to study the Jaffe complexes prepared by different concentrations of aqueous creatinine solutions in vitro, Gangopadhyay et al. used surface‐enhanced Raman scattering, fluorescence, and ultraviolet spectroscopy. By observing the intensity of the surface‐enhanced Raman scattering signature for creatinine in the Jaffe complex prepared from the solution, the concentration in any solution can be determined in vitro with the help of this plot up to 0.3 mg/dL.^151^

The colorimetric method is an optical technique in which the color variations that occur in the sample due to chemical reactions are measured. This change in color can be due to creatinine reactions with enzymes,^152^, ^153^ NP,^35^, ^48^, ^49^, ^154^ or other chemical reactions.^29^ Color variation is detectable by spectral examination at one or more specific wavelengths in the visual range using a spectrophotometer or other color processing methods.^23^ Using smartphones for imaging and processing color changes caused by the creatinine reaction with other materials are new and practical methods for measuring creatinine. Fu et al. presented a quick method with an integrated system, including a paper‐based chip and a smart detection device (smartphone) to determine the human SCr concentration based on Jaffe reaction theory. The detection limit and linear range of this device are 0.08 mM and 0.2‐1 mM, respectively.^155^ The advantages of this method are simple, inexpensive, high‐speed detection, and good accuracy. The interference of other materials in the chemical reaction and the disturbing color change in the sample is one of the most fundamental problems in this method.

MS is also a powerful analytical technique in which the sample is converted into gaseous ions (with or without the component), and then the mass to charge ratio (*m/z*) and its relative abundances are determined. A mass spectrum is a plot of the ion signal as a function of the *m/z*. MS is a sensitive, rapid, and selective method for the determination of creatinine.^156^ Takahashi et al. and Hušková et al. used this method to measure creatinine.^156^, ^157^

The results of these methods for the determination of creatinine are presented in Table 5.

**TABLE 5:** The results of using the spectroscopic and colorimetric methods for the detection of creatinine

Measurement methods	Wavelength (maximum absorbance)	Linear/calibration range	LOD	Refs.
Spectrometry	530 nm	4.4‐620 μM	145 nM	^17^
Mass spectrophotometry	–	0.25‐2.0 mg/mL (2.2‐17.7 mM)	0.4 μg/mL (3.5 μM)	^158^
Optoelectronic detector	465 nm	40‐4000μM	22 μM	^159^
Smart detection device	498 nm	10‐300 μM	3 μM	^159^
Spectrophotometric	–	0.2‐1 mM	0.08 mM	^155^
Spectrophotometric	500 nm	0.01‐1 μM	8.4 nM	^145^
Tandem mass spectrometry	A670/A403	0‐4.2 μM	53.4 nM	^41^
spectroscopy	–	0.06‐60 mM	0.2 μM	^156^
Surface‐enhanced Raman	785 nm(Raman peak: 649 vs. 616 cm–1)	–	10 mg/dL (0.88 mM)	^149^
Raman spectroscopy		Up to 1 μM	24.8 nM	^160^
Colorimetric	G (green) and B (blue) value	0.19‐7.64 mg/dL (1.68‐67.5 μM)	0.19 mg/dL (1.68 μM)	^23^
Spectrophotometric	A600/520	15‐40 mg L^−1^ (133‐354 μM)	13.7 mg L^−1^ (121 μM)	^98^
Photometric	A520/398	0.01‐1 μM	12 nM	^22^
colorimetric	*A*619/*A*522	10‐100 nM	2.16 nM	^161^
Spectrophotometric	A560/390	0.01‐1 μM	3.0 nM	^49^
Colorimetric	A 670/ 520	0.1‐20 mM	80 μM	^48^

### 2.7 Chemiluminescence‐based sensors

**FIGURE 7:**
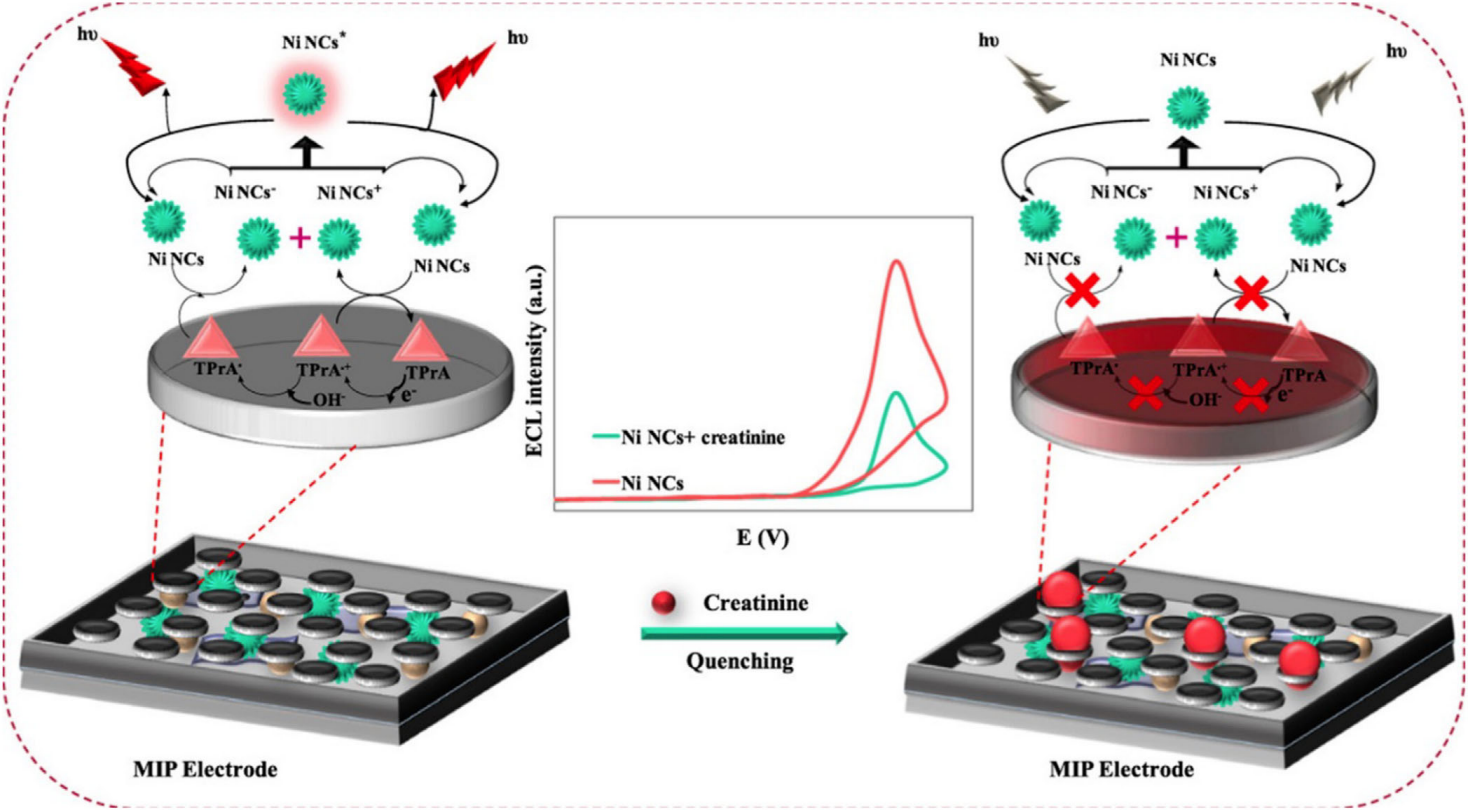
Illustration of nickel nanoclusters as a new emitter for MIP electrochemiluminescence‐based sensor toward nanomolar detection of creatinine.^115^

In Luminescence based sensors, emission of light by certain materials when they are relatively cool. Luminescence emission occurs after an appropriate material has absorbed energy from a source such as chemical reactions (not resulting from heat). The energy lifts the atoms of the material into an excited state, and then, because excited states are unstable, the material undergoes another transition, back to its unexcited ground state, and the absorbed energy is liberated in the form of either light or heat or both. If the molecule is stimulated by a chemical reaction, it is called chemiluminescence. The application of the chemiluminescence system for the determination of creatinine is investigated. In one of these studies, creatinine reacted with $H_{2}O_{2}$, which produces a strong chemiluminescence signal. Also, $Co^{2+}$ can catalyze the reaction of $H_{2}O_{2}$ and creatinine efficiently (chemiluminescence intensity enhances 24 times). After the return of the excited state species to the ground state, light is generated. For this method, the linear/calibration range is between 0.1 and 30 μM and the detection range is 72 nM.^36^ Electrochemiluminescence is a type of chemiluminescence in which the reaction that generates light starts with an electrical current and ends with it) because of luminescence instability (. In the electrochemiluminescence, the energy stimulation factor is an electrochemical reaction. Electrochemiluminescence with MIP is also used to measure of creatinine by Babamiri et al. This method has accurate and ultrasensitive analytical performance for creatinine measurements. The linear/calibration range and the LOD are 5‐1 mM and 0.5 nM to determine the creatinine concentration (Figure 7).^115^

The ideal performance of chemiluminescence‐based sensors with sensitive and accurate detection has a great advantage, but the costly procedure and restricted availability of the device, kits, and solutions limit the application of this procedure according to the manufacturing company trends.

## 3 DISCUSSION AND CONCLUSION

**FIGURE 8:**
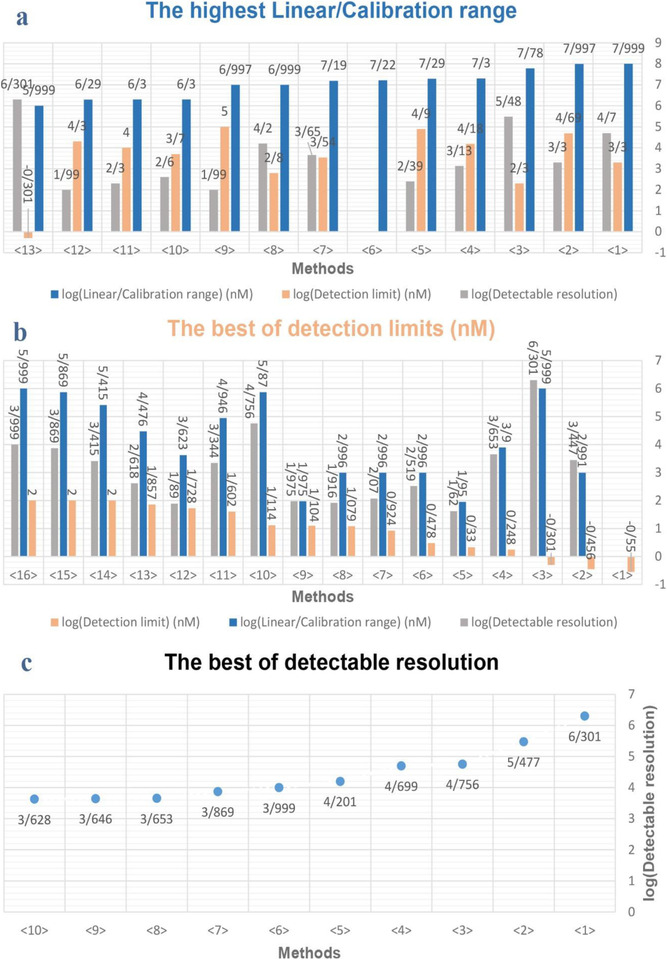
(A) Comparison of the highest linear/calibration range of different methods. Values were calculated by log. Log (linear/calibration range) shows the difference between the highest and lowest linear/calibration range in each method (in terms of logarithm and nanomolar scale)—< 1 > :[1] ‐ < 2 > :[2] ‐ < 3 > :[3] ‐ < 4 > :[7] ‐ < 5 > :[8] ‐ < 6 > :[11] ‐ < 7 > :[16] ‐ < 8 > :[19] ‐ < 9 > :[20] ‐ < 10 > :[21] ‐ < 11 > :[23] ‐ < 12 > :[26] ‐ < 13 > :[27]. (B) Comparison of the best of detection limits in different methods. Values were calculated by log. For example:(lg(0.28) = −0.55)—< 1 > :[29] ‐ < 2 > :[31] ‐ < 3 > :[27] ‐ < 4 > :[37] ‐ < 5 > :[38] ‐ < 6 > :[42] ‐ < 7 > :[43] ‐ < 8 > :[39] ‐ < 9 > :[47] ‐ < 10 > :[25] ‐ < 11 > :[49] ‐ < 12 > :[50] ‐ < 13 > :[53] ‐ < 14 > :[54] ‐ < 15 > :[60] ‐ < 16 > :[61]. (C) Detectable resolution is the result of linear/calibration range's division into detection limit. Values were calculated by log. As shown in the figure, a tandem mass spectrometry method has the highest detectable resolution with 299,700 measurable points (log(299,700) = 5/477)—< 1 > :[27] ‐ < 2 > :[3] ‐ < 3 > :[25] ‐ < 4 > :[1] ‐ < 5 > :[19] ‐ < 6 > :[61] ‐ < 7 > :[60] ‐ < 8 > :[37] ‐ < 9 > :[16] ‐ < 10 > :[32].

Creatinine is a nontoxic and unnecessary substance for biometabolism, which indicates a great contribution to the monitoring of kidney biofunction. Due to reported limitations, including specificity, sensitivity, interference errors, reaction time, accuracy, linear/calibration range, detection limit, real‐time measurement, cost, and application, emerging methods and biosensors for creatinine are described. Most of the tests that are done to determine creatinine are performed by creatinine reaction with another biomarker (enzyme, etc.). These reactions occur under certain pH and temperature conditions to make a reaction between them. Extremely low or high levels of pH cause disruption of the activity of most enzymes. Also, one of the factors in the stability of enzymes is pH and there is a region of pH optimal stability for each enzyme activity.^162^ In addition to the effect of the reaction process,^163^ temperature also affects the pH measurements. Molecular vibrations, which increase as the temperature rises, cause ionization of water and the formation of hydrogen ions and reduce the amount of pH. So the pH value of a solution is dependent on the temperature. In addition to pH and temperature, there are other factors, such as ionic strength,^164^ which can affect the reactions. Each of these chemical and physical parameters must be optimized and considered for reactions to be reproducible and accurate. It is difficult to directly determine the concentration of creatinine in the body due to the presence of interfering factors; therefore, various receptors (commonly enzyme‐based) are used to boost the efficiency of detection. Enzymes indicate great accuracy and sensitivity, with low rate of interfering capability. However, the high cost, stability, temperature and pH dependence, substrate concentration, and enzyme activity are the main limitation for enzymes application.^32^ MIP is commonly applied in sensor designing, and one of the most important factors in MIP is the choice of a functional monomer, which fits with the template molecule. Cost effectiveness, relative simplicity, higher selectivity, and great stability and sensitivity of this method are highly regarded. Today, NPs are also used along with other methods due to its high absorption capacity and large S‐V ratio. Spectrometry and colorimetry by using chemical reactions and NPs are the simplest methods for determining creatinine. Also, the reaction of enzymes and creatinine antibodies cause alternation in absorption ratio and solution color. Electrochemical methods have been reported based on potential, current, and conductivity changes in the sample; the small size and high accuracy are the advantages of this method. Various ISFETs and electrodes are designed that are added daily to their numbers. However, chromatographic methods have been used less than other methods due to their low response rate and multistage procedure (spectrophotometric, etc.). But this method offers a much higher detection limit and accuracy.^69^ Besides, chemiluminescence‐based sensors provide accurate and ultrasensitive analytical performance toward the detection of creatinine. The costly procedure and restricted availability of the device, kits, and solutions limit the application of this procedure according to the manufacturing company trends. As stated above, the linear/calibration range and LOD are the most important characteristics of a sensor to determine the concentration of creatinine. Figure 8 compares the highest linear/calibration range of different methods. In this figure, the results of the methods are also presented in detectable resolution for better comparison. Based on the wide detection range and difference in intervals, the logarithmic approach is applied to represent the values.

The design of the point‐of‐care testing (POCT) (at a lower cost) is of great importance to improve patient health monitoring and life quality.^144^ POCT should provide ideally, reliable quantitative results, comprehensible presentation, simple decision support, and connectivity to other information systems, including the patient's electronic health record.^165^ It seems that the use of colorimetric methods using a smartphone is the easiest way to detect creatinine concentrations. But this method is not very accurate at present and requires a lot of studies.^23^, ^166^ The goal of all these researches is to provide a new method for building up a small biosensor that is used by the patient remedies. This sensor should include features such as high accuracy, acceptable linear/calibration range and detection limit, good stability, reasonable cost, reliable, less sample volume, noninvasive, higher response rate, and easier to use.

## CONFLICT OF INTEREST

The authors declare that they have conflict of interest.

## AUTHOR CONTRIBUTIONS

Conceptualization: Mahdad  Esmaeili and Ahmad Mobed. Writing the manuscript and provided data: Ramin Narimani, Ahmad Mobed, and Mahdad  Esmaeili. Edit and review: Hamid Tayebi Khosroshahi and Seyed Hossein Rasta. All authors reviewed the final manuscript.
